# Colonization of xenograft tumors by oncolytic vaccinia virus (VACV) results in enhanced tumor killing due to the involvement of myeloid cells

**DOI:** 10.1186/s12967-016-1096-1

**Published:** 2016-12-20

**Authors:** Mehmet Okyay Kilinc, Klaas Ehrig, Maysam Pessian, Boris R. Minev, Aladar A. Szalay

**Affiliations:** 1 Department of Biochemistry, Biocenter, University of Würzburg, Am Hubland, 97074 Würzburg, Germany; 2 San Diego Science Center, Genelux Corporation, San Diego, CA USA; 3 Department of Radiation Medicine and Applied Sciences, Rebecca & John Moores Comprehensive Cancer Center, University of California, San Diego, CA USA

**Keywords:** MDSCs, VACV, iNOS, Oncolytic virus therapy, NO, Innate immune system, Antitumor immune response, Antiviral immunity

## Abstract

**Background:**

The mechanisms by which vaccinia virus (VACV) interacts with the innate immune components are complex and involve different mechanisms. iNOS-mediated NO production by myeloid cells is one of the central antiviral mechanisms and this study aims to investigate specifically whether iNOS-mediated NO production by myeloid cells, is involved in tumor eradication following the virus treatment.

**Methods:**

Human colon adenocarcinoma (HCT-116) xenograft tumors were infected by VACV. Infiltration of iNOS^+^ myeloid cell population into the tumor, and virus titer was monitored following the treatment. Single-cell suspensions were stained for qualitative and quantitative flow analysis. The effect of different myeloid cell subsets on tumor growth and colonization were investigated by depletion studies. Finally, in vitro culture experiments were carried out to study NO production and tumor cell killing. Student’s *t* test was used for comparison between groups in all of the experiments.

**Results:**

Infection of human colon adenocarcinoma (HCT-116) xenograft tumors by VACV has led to recruitment of many CD11b^+^ ly6G^+^ myeloid-derived suppressor cells (MDSCs), with enhanced iNOS expression in the tumors, and to an increased intratumoral virus titer between days 7 and 10 post-VACV therapy. In parallel, both single and multiple rounds of iNOS-producing cell depletions caused very rapid tumor growth within the same period after virus injection, indicating that VACV-induced iNOS^+^ MDSCs could be an important antitumor effector component. A continuous blockade of iNOS by its specific inhibitor, L-NIL, showed similar tumor growth enhancement 7–10 days post-infection. Finally, spleen-derived iNOS^+^ MDSCs isolated from virus-injected tumor bearing mice produced higher amounts of NO and effectively killed HCT-116 cells in in vitro transwell experiments.

**Conclusions:**

We initially hypothesized that NO could be one of the factors that limits active spreading of the virus in the cancerous tissue. In contrast to our initial hypothesis, we observed that PMN-MDSCs were the main producer of NO through iNOS and NO provided a beneficial antitumor effect, The results strongly support an important novel role for VACV infection in the tumor microenvironment. VACV convert tumor-promoting MDSCs into tumor-killing cells by inducing higher NO production.

## Background

The mechanisms by which vaccinia virus (VACV) interacts with the innate immune components may play a decisive role in its antitumor activity by tilting the immune response from viral clearance to tumor elimination. The inherent ability to rapidly replicate in, and lyse human tumor cells in comparison with other viruses as well as its large foreign gene-carrying capacity make VACV a leading candidate for the use in cancer therapy [1]. Until now, preclinical and clinical studies have demonstrated that various VACVs have a broad spectrum of anticancer activity and good safety [2]. Tumor-targeting mechanisms of VACV include virus-mediated direct oncolysis, antivascular effects and induction of antitumor immune responses [3–5]. The latter mechanism of action might be essential in the elimination of tumor cells which are able to escape virus infection [6]. The three critical stages to ensure the effectiveness of any oncolytic virus therapy include: efficient virus targeting to tumor sites, fast and continuous virus replication in tumor cells, and resistance to the host antiviral immunity. Virus elimination by the host immune system is a major obstacle to the oncolytic virus therapy. An important question that remains to be answered is whether the host immune system, adept at controlling viral infections, would also have an impact on the tumor.

The tumor microenvironment presents a niche, which supports the proliferation of malignant cells while promoting the evasion of immune surveillance [7]. The recruitment of regulatory/suppressor immune cells like regulatory T cells, and myeloid-derived suppressor cells (MDSCs) ultimately enhances the pro-tumorigenic and suppressive nature of the microenvironment. MDSCs, which are induced by tumor-derived inflammatory factors, are a heterogeneous population of immature myeloid cells. They constitute a major part of the tumor-infiltrating immune cells and play a central role in the regulation of the immune system [8]. In mice, they are characterized by the expression of CD11b and Gr-1. Anti-Gr-1 antibody, which binds to the myeloid differentiation marker Gr-1, recognizes two epitopes, Ly6G and Ly6C. The classification made based on these markers initially revealed two main subsets of MDSC. The CD11b^+^ Ly6G^+^ Ly6C^lo^ (PMN-) MDSC subset displays a granulocytic, polymorphonuclear phenotype, while the CD11b^+^ Ly6G^−^ Ly6C^hi^ subset exhibits a mononuclear phenotype (MO-) [9]. Lately, MDSCs have been categorized into other different subsets [10]. Extensive studies have shown that MDSCs accumulate at tumor sites, suppress the antitumor immune response and promote tumor progression [11]. MDSCs use a variety of mechanisms depending on different immune-regulators such as inducible nitric oxide synthase (iNOS), arginase, reactive oxygen species (ROS), and TGF-β. Among them, iNOS is expressed in high amounts and can act as a powerful modulator in different cancer-related events including apoptosis, angiogenesis, cell cycle, invasion, and metastasis due to its substantial NO production [12]. Although the role of NO in mediating immune system such as inhibiting T cell proliferation or suppressing their function is very well documented, its effect on tumor cells remains controversial; In contrast to tumoricidal effects, NO has also been reported to have tumor promoting effects [13, 14]. The concentration and timing of the NO accumulation has been implicated in its dichotomous effects [15]. Therefore, understanding the role of iNOS^+^ cells in any anticancer approach will help in improving cancer treatment strategies.

The interaction of oncolytic viruses with myeloid cell components of the innate immune system has only recently become an important research endeavor. Recent reports have demonstrated the important role of myeloid cells in virus delivery and oncolytic therapy when macrophages or MDSCs were used as transport vehicles [16, 17]. Furthermore, MDSCs have an effect on the function of natural killer (NK) cells, when oncolytic viruses are introduced [18–20]. Lately, it has been proposed that VACV can act as an immunomodulator, triggering an inflammation that can vanquish tumor driven protective mechanisms [4–21]. Virus infection at the tumor site leads to cell death, the release of danger signals, tumor antigens and inflammatory cytokines, which can overcome an established immunosuppressive microenvironment, subsequently initiating antitumor immune responses. We recently reported that colonization of human colorectal cancer xenografts with the oncolytic VACV strain GLV-1h68 in nude mice, is followed by significant upregulation of murine proinflammatory cytokines and chemokines such as interferon-gamma (IFNγ), IFNγ-induced protein 10 (IP-10), monocyte chemoattractant protein (MCP)-1/3/5, macrophage inflammatory protein 1 (MIP-1), regulated on activation normal T cell expressed and secreted (RANTES), IL-6, IL-1b and tumor necrosis factors (TNF-α), as well as infiltration of F4/80^low^ CXCR4^+^ myeloid cells [22]. This finding is in accordance with previous reports of different tumor models [3, 23]. While virus-mediated inflammation may lead to antitumor immunity, this response might also target viral vectors, limiting their therapeutic efficacy. One of the key elements of the antiviral response is iNOS, which is expressed by myeloid cells. iNOS-producing M1 macrophages have been particularly shown to mediate the innate immune defense against viruses in infection models [24, 25]. However, how iNOS^+^ MDSCs could modulate the antitumor immune response during an infection of tumor tissue remains largely unknown. Mechanistic studies exploiting the interaction of the host immune system with the oncolytic viruses are vital in understanding the therapeutic potential of VACV. Herein we report an extensive accumulation of iNOS^+^ MDSCs at VACV-infected tumor sites. In vivo depletion of this cell subset or blocking its function promoted tumor growth demonstrating the iNOS^+^ MDSCs’ potential therapeutic benefits. We further confirmed that a higher amount of NO production was responsible for synergistic tumor cell killing by VACV and MDSCs.

## Methods

### Cell culture, mice, tumor induction

Human colon carcinoma cells HCT-116 and African green monkey fibroblasts (CV-1) cells were cultured as described before [22]. Five- to six-week old male Hsd:athymic Nude-Foxn1nu mice (Harlan, Indianapolis, IN) were implanted subcutaneously (s.c.) with 5 × 10^6^ HCT-116 cells (in 100 μL PBS) into the right hind leg. Tumors were allowed to reach a size of 250–350 mm^3^ before treatment. All studies were approved by the Institutional Animal Care and Use Committee of Explora Biolabs (San Diego Science Center, protocol number EB11-025,CA).

### Virus and reagents

LIVP 1.1.1 is a less virulent wild-type isolate of a strongly replicating LIVP strain as described before [26]. TurboFP635 (aka Katushka) is a far-red fluorescent protein from sea anemone *Entacmaea quadricolor* [27]. For the generation of the GLV-2b372 from LIVP 1.1.1, the cDNA encoding for Katushka was PCR-amplified using the plasmid FUKW (kindly provided by Dr. Marco J. Herold, University of Würzburg, Würzburg, Germany) as a template with primers FUKW-5 (5′-GTCGACCACCATGGTGGGTGAGGATAGCGTGC-3′) and FUKW-3 (5′-TTAATTAATCAGCTGTGCCCCAGTTTGC-3′). The PCR product was gel-purified and cloned into the pCRII-Blunt-TOPO^®^ vector (Life Technologies, Carlsbad, CA) and then released by enzymatic *Pac*I/*Sal*I (New England Biolabs, Ipswich, MA) digestion. Subsequently, the cDNA fragment was subcloned into the vaccinia transfer vector for the J2R (TK) locus, placing TurboFP635 under control of the vaccinia synthetic early/late (SEL) promoter. The resulting plasmid construct pTK-SEL-TurboFP635 was sequence confirmed and used for the construction of GLV-2b372 using guanine phosphoribosyltransferase selection [28]. LIVP 1.1.1, or GLV-2b372, GLV-1h68 were administered systemically to HCT-116 tumor-bearing animals by retro-orbital (r.o.) injection of 2 × 10^6^ plaque-forming units (PFU) in 100 μL PBS on day 0. Control mice were injected with 100 μL PBS. For monocytic or granulocytic cells depletion, animals received single vs. continuous (twice per week) intraperitoneal (i.p.) injections of clodronate liposomes (2 mg) (Encapsula NanoSciences, Brentwood, TN) or 1A8 rat mAb (1 mg) (BioXCell, West Lebanon, NH), respectively. For inhibition of iNOS activity, N6-(1-iminoethyl)-l-lysine dihydrochloride (L-NIL) was injected i.p. initially at a dose of 0.2 mg/100 μL PBS followed by 0.1 mg every other day. Nanoparticles were formulated as previously described with minor modifications [29]. In brief, 30 mg PLGA and 2 mg of LPS (Sigma, St. Louis, MS) in 1 mL of chloroform was emulsified in 6 mL of 2% PVA to form an oil-in-water emulsion. The emulsification was carried out using a micro-tip probe ultrasonic sonicator set at 55 W of energy output (XL 2015 Sonicator^®^ ultrasonic processor; Misonix, Inc, Farmingdale, NY) for 2 min over an ice bath. The emulsion was stirred overnight on a magnetic stir plate to allow evaporation of chloroform and formation of PLGA-NPs. PLGA-NPs were recovered by ultracentrifugation at 30,000 rpm for 30 min at 4 °C (Beckman OptimaTM LE-80K, Beckman Instruments, Pasadena, CA), washed twice with sterile nano water to remove PVA and then lyophilized for 48 h (VirTis Company, Freeze Dryer, Stone Ridge, NY). An analysis of the LPS nanoparticles was made using the EndoZyme Kit (Hyglos GmbH, Germany), using manufacturer’s protocol. The concentration was 7.3 μg of LPS/mL of nanoparticles.

### Preparation of single cell suspensions, enrichment and fluorescence-activated cell sorting (FACS)

Mice were followed for a period of up to 21 days post-infection (d.p.i.) and were sacrificed at specific time points (1, 3, 7, 14, 21 d.p.i). Single-cell suspensions from tumors and spleen were prepared essentially by enzymatic digestion as previously described [2]. Flow cytometric analysis was performed on a FACSCanto flow cytometer (BD Pharmingen, San Jose, CA) at VA hospital Flow Cytometry Core facility using established protocols. Flow analysis, gating strategy and determination of the absolute number regarding the cell population in interest were explained in detail in our previous publication [30]. Fluorochrome-conjugated anti-mouse monoclonal Antibodies (mAbs) to iNOS (6/iNOS/NOS type II), CD11b (M1/70), Ly6G (1A8), CD45 (30-F11), and all isotype controls were purchased from BD Pharmingen. F4/80 (BM8) was obtained from eBioscience, San Diego, CA. Intracellular iNOS staining was as described previously [13].

### Vaccinia viral titers

Tumors were excised and placed in 500 μL of PBS supplemented with Complete Protease Inhibitor Cocktail (Roche Diagnostics, Indianapolis, IN). Samples were then homogenized using a MagNA Lyser (Roche Diagnostics) at a speed of 5000 rpm for 30 s. Following three subsequent freeze–thaw cycles (−80 °C/37 °C water bath), tumor supernatants were collected by centrifugation (600*g*, 5 min, RT). Viral titers in tumor supernatants were measured by standard plaque assay on 24-well plates of confluent CV-1 cells, with all samples assessed in duplicate, as described previously [22].

### In vivo fluorescence imaging of TurboFP635

In vivo fluorescence imaging and analysis was performed using a Carestream animal imager (excitation, 590 nm; emission, 670 nm) using the Molecular Imaging Second Edition software (Carestream Health, Rochester, NY). Background fluorescence was subtracted and data were presented as mean relative fluorescent units (RFU) per tumor area.

### In vitro culture experiments

To purify iNOS^+^ cells, single-cell suspensions from spleen were magnetically labeled with anti-Ly-6G microBeads according to the manufacturer instructions and then the cell suspension was loaded onto autoMACS in order to enrich Ly-6G^**+**^ cells (Miltenyi Biotec, Cologne, Germany). The purity of the total iNOS^+^ population was typically higher than 90%. Isolated and combined iNOS^+^ effector cells (from 3 to 4 mice per group) were placed into the upper chamber of 96-well Transwells (0.4 μm pore size membrane; Corning, NY) at different E:T ratios. The lower chamber of the Transwells contained 5000 HCT-116 target cells plated overnight. Coculture was conducted in MLR media [DMEM plus 5% FBS with 10 mM HEPES (pH 7.4), 1% sodium pyruvate, 1% penicillin/streptomycin, 1% l-glutamine, 0.4% l-arginine HCl, 1% folic acid/l-asparagine, and 0.2% 2-ME] supplemented with recombinant Murine IFN-γ (20 ng/mL, Peprotech, Rocky Hill, NJ) and the specific killing of target cells was tested by using Alamar Blue (Invitrogen, Waltham, MA) which is added at 10% of the sample volume followed by 4–16 h incubation. Every 4 h the resulting Alamar blue fluorescence was read on SpectraMax M5 reader (Molecular Devices, Sunnyvale, CA) with excitation at 540 nm and emission, 590 nm. The percentage of lysis was calculated using the formula; %Lysis = 100 × {[(AF of targets alone)] − [(AF of mix) − (AF of effectors alone)]}/{AF of targets alone} where AF is the mean fluorescence for the triplicate wells after the mean fluorescence of the wells containing medium alone was subtracted. To block NO production in some wells, L-NIL at a final concentration of 0.5 mM was added. In another experimental setup, Annexin V Apoptosis Detection Kit I was used for viability staining according to the manufacturer instructions (BD Pharmingen) and culture supernatant was collected for NO measurement. Nitrite assay was performed as described before [30].

### Statistical analysis

Student’s *t* test was used for comparison between groups in all of the experiments. In all analyses, *p*  ≤  0.05 was considered significant.

## Results

### Robust increase of tumor-infiltrating iNOS^+^ myeloid cells during viral infection

**Fig. 1:**
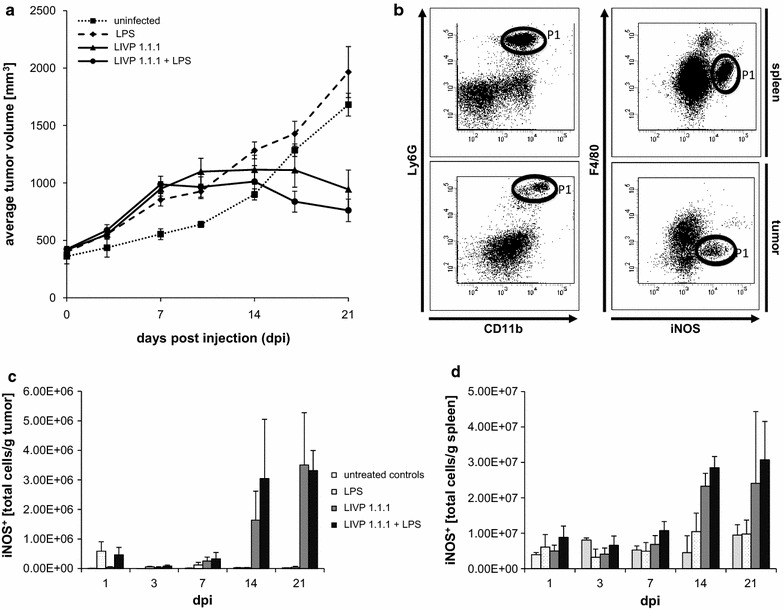
Effect of VACV on tumor growth and post-therapy intratumoral PMN-MDSC kinetics. **a** Growth of HCT-116 tumors in LIVP 1.1.1 and control-treated mice. Tumor-bearing nude mice were treated with a single injection of LIVP 1.1.1 or LPS-nanoparticles or combination of both or PBS alone (uninfected). Tumor size was measured twice a week. Data shown are representative of two independent experiments with similar results. *Error bars* indicate SD (n = 3–5 mice/group). **b** Pre-treatment flow cytometry analysis of PMN-MDSCs in both tumor and spleen. A major iNOS^**+**^ cell subset with a phenotype of CD11b^+^ly6G^+^F4/80^low^ was detected in single cell suspensions from both tumor and spleen. FACS plots are shown with the population of interest (PMN-MDSCs) in *bold circles*. **c**, **d** Accumulation kinetics of PMN-MDSC upon VACV treatment. The absolute numbers of PMN-MDSCs were determined by flow cytometry. Results are expressed as the average number of cells per gram of tissue and were obtained from three separate experiments with 3–5 mice. *Error bars* represent mean and SD. The differences between the VACV alone treated group (*dark gray bar*) and untreated controls (*light gray bar*) were significant for day 14 (tumor; *p* = 0.014 and spleen; *p* = 0.02) and for day 21 (tumor; *p* = 0.006). *Black bars* and *light gray bars* represent LIVP 1.1.1 + LPS-nanoparticles combination and untreated controls respectively

We first monitored the tumor growth and the infiltration of iNOS^+^ myeloid cell population into HCT-116 tumors after treatment. Starting from day 10-post virus injection, tumor growth stopped in LIVP 1.1.1-treated animals and entered a steady-state phase followed by regression while control tumors continued to grow (Fig. 1a). In order to study the accumulation kinetics of iNOS^+^ subsets on day 1, 3, 7, 14, and 21 following the treatment, single-cell suspensions from spleen and primary tumors were stained for Ly6G, CD11b and F4/80 and iNOS. Homogeneously stained single population of CD11b^+^ly6G^+^F4/80^low^ cell subset expressing iNOS was detected both in spleens and tumors (Fig. 1b). The phenotype of the tumor-infiltrating myeloid cells resembled PMN-MDSCs described previously by us and others [9, 30]. Since LPS is well known to induce iNOS expression [31], LPS-containing nanoparticles, were used as a positive control in these experiments. The absolute number of intratumoral MDSC expressing iNOS increased rapidly within 24 h, and subsequently dropped in LPS-nanoparticle-treated alone (white and black-dotted bar) as well as LPS-nanoparticle + LIVP 1.1.1 combination-treated groups (black bar). The accumulation of PMN-MDSCs in a single dose LIVP 1.1.1 injection group however was delayed until day 7 post-treatment, followed by the most drastic change with an average of 72-fold increase between days 7 and 14 (Fig. 1c). Combination treatment resulted in the same kinetics; although it is interesting to note that this group recruited the highest amount of intratumoral iNOS^+^ PMN-MDSCs on day 14, which correlated with slightly improved tumor regression. Furthermore, the difference in tumor size between the combination therapy group and control (PBS or LPS alone) groups reached a statistically significant level on day 21 (*p* < 0.0001). Analysis of the iNOS^+^ PMN-MDSCs infiltration kinetics in the spleen revealed that accumulation of these cells followed the same trend observed in the tumor. A statistically significant increase for their average absolute number among different groups was not detected before day 7. Thereafter iNOS^+^ PMN-MDSCs populated the spleen with a significant fourfold increase on day 14 (*p* = 0.02) which did persist, but not significantly, until 21 days postinfection (dpi) in both of the virus-treated groups.

The lack of immunocompetent models for investigating therapies with our VACV constructs required us to establish xenograft tumor models in which tumor cells could support viral replication in spite of fully intact innate immunity. Like many other oncolytic viruses, vaccinia virus replication is considered to be species-specific and similarly, our VACV constructs replicate well in human tumors, with poor replication seen in most murine tumor cell lines. Nevertheless, the elevated level of Gr1^+^CD11b^+^ cell infiltration were observed in many other different models such as vesicular stomatitis virus (VSV) in melanoma and mesothelioma, Western Reserve strain VV (Vvdd) in AT-3, Reovirus in ovarian cancer pointing out these cells may play an important role in anti-VACV immune response [18, 32–34].

### Tumor regression coincides with the accumulation of PMN-MDSCs and enhanced iNOS expression

**Fig. 2:**
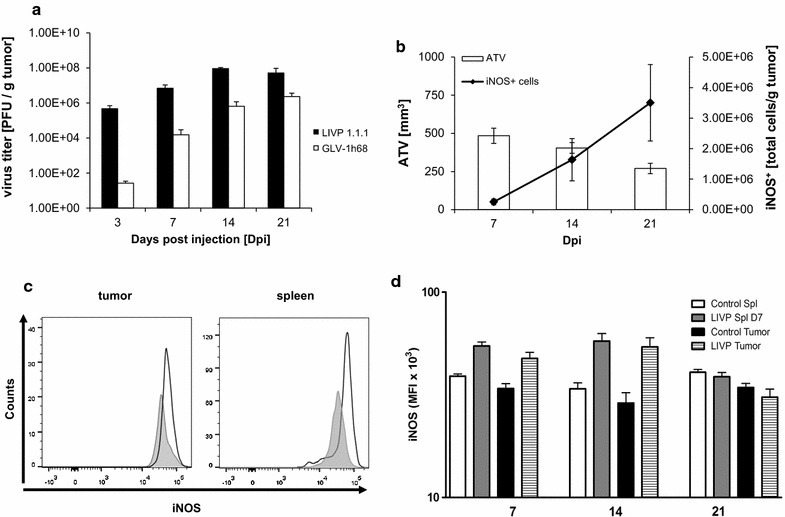
Result of iNOS expression on virus titer and tumor volume. **a** HCT-116 tumors were harvested 3, 7, 14 and 21 dpi and infectious viral particles were quantitated on CV-1 (*n* = 3–5 mice/time point/experiment). The bar graph shows the mean virus titer as PFU per gram of tumor for both LIVP 1.1.1 and GLV-1h68 as test versus control virus (GLV-1h68 is a recombinant isolate from the VACV LIVP strain, as described previously [41]), respectively. **b** An inverse relationship between the average tumor volume and the number of iNOS^+^ cells induced by LIVP 1.1.1. The plot represents the infiltration kinetics of iNOS^+^ cells (*solid line*) over change in tumor growth (*bars*) on days 7, 14 and 21. **c** iNOS expression was assessed as relative MFI using flow cytometry. Histogram shows relative MFI on gated iNOS^+^ PMN cells isolated from the tumor and spleen on day 7. **d** The change of iNOS MFI over time. All of the treatment groups have a significant difference from the control except for the day 21; day 7 tumor; *p* = 0.0174 and spleen; *p* = 0.0035 and day 14 tumor; *p* = 0.0079 and spleen; *p* = 0.0052). Each time point was repeated at least two times

Next, we sought to determine whether the prevalence of the treatment-induced PMN-MDSC subset correlated with tumor growth and/or controlled viral load. Initially, we hypothesized that excessive iNOS^**+**^-cell migration into the tumor in response to viral infection would cause viral clearance. Earlier studies reported that nitric oxide production is a key effector factor in antiviral activities against VACV [35, 36]. Recent reports however suggested the opposite and indicated that NO is not essential for the control of VACV’s replication or dissemination [37, 38]. To determine the virus infection kinetic in tumors, mice treated with LIVP 1.1.1 and 1h68 were sacrificed on 3, 7, 14 and 21 dpi and tumor tissues were homogenized. Figure 2a shows the results of a viral titer analysis as pfu/gram of tumor. Three days after LIVP 1.1.1 injection, 4.6 × 10^5^ ± 1.4 × 10^5^ viral pfu were detected in the tumor (Fig. 2a). The exponential increase in LIVP 1.1.1 titer reached its maximum at a two-log fold level on day 14 postinfection. Consistent with our previous data [22], viral colonization pattern for 1h68 followed identical trend as of LIVP 1.1.1 except that a significantly lower titer compared to primary LIVP 1.1.1 tumors was observed at all-time points. As the tumor growth started to decrease between days 7 and 14, VACV particles along with the actual number of iNOS^+^ cells in the tumor started to increase indicating an inverse relationship between the average tumor volume (ATV) and iNOS^+^ cells induced by the virus (Fig. 2b). Moreover, the gradual increase in the viral replication and infection of the tumor induced an increase in the expression of iNOS as detected by mean fluorescence Intensity (MFI) starting from day 7. A higher iNOS expression by PMN-MDSC was observed in the tumor (47.4 × 10^3^ ± 6.9 × 10^3^) compared to the level in the spleen (38.9 × 10^3^ ± 1.8 × 10^3^) as shown in the histogram (Fig. 2c). The following figure, presents the quantification of this signal. The intensity of MFI level stayed constant till day 14 but leveled off on day 21. Overall, our results suggested that NO might play an important role in the VACV-induced pathogenesis by enhancing the suppression of tumor growth without affecting the viral load.

### Depletion of different myeloid cell subsets causes diverse effects on intratumoral VACV colonization and tumor growth

**Fig. 3:**
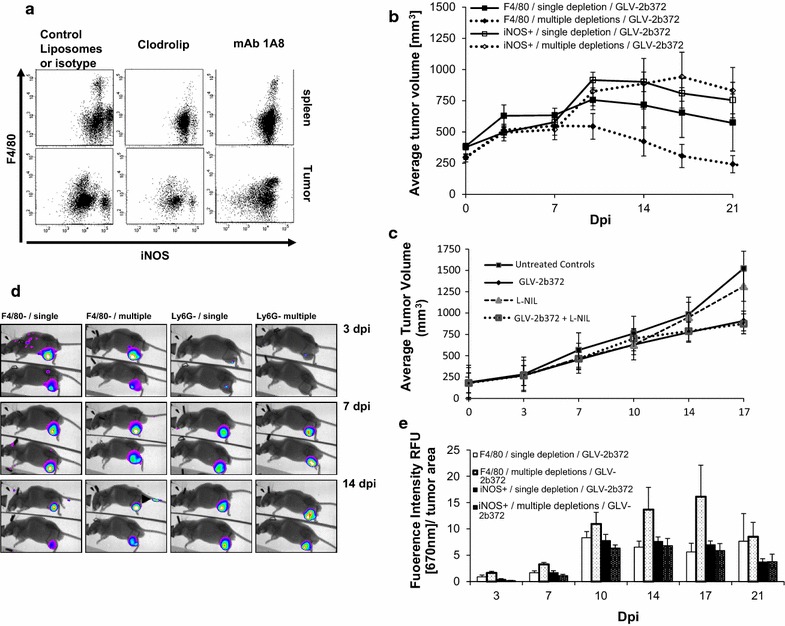
Targeting different myeloid cell subsets. **a** A representative FACS dot plot shows the results of depletion by mAb 1A8 or clodrosome. iNOS^+^ neutrophilic (Neu) and F4/80^+^ monocytic cells (Mac) were depleted to different extents 48 h. post-depletion. **b**, **c** Depletion or blocking of iNOS^+^ Neutrophilic cells showed a similar result on tumor growth between days 7 and 10 post-VACV infection. The effect of single or multiple-depletion of iNOS^+^ neutrophilic (Neu) versus F4/80^+^ monocytic cells (Mac) on tumor size in **b** was compared to the iNOS-blocking agent, L-NIL in **c**. **d**, **e** Monitoring of tumor growth by fluorescence imaging. Carestream imaging system performed visualization of viral tumor colonization and the signal intensity was quantified as mean RFU per tumor area. The results shown are from one of two independent experiments that produced similar results

To evaluate the significance of Ly6G^+^ iNOS^+^ MDSCs accumulation in the tumor following VACV infection, we next carried out depletion studies. We tested both anti-Ly6G antibody (mAb 1A8) and clodronate encapsulated liposomes (Clodrosome) to particularly deplete Ly6G^high^ iNOS^+^-MDSCs. A single injection of 1 mg of 1A8 2 days prior to virus treatment resulted in more than 95% depletion of iNOS^+^ cells in both spleen and the tumor as compared to isotype control treated samples and detected by their total cell number counts. Mice administered with Clodrosome (2 mg) showed a substantial reduction in the number of both the splenic and intratumoral F4/80^+^ cells as compared to empty liposomes, whereas the reduction of iNOS^+^ cell number in the spleen and tumors remained at the level of 46 and 32%, respectively. Figure 3a shows a representative dot plot of mouse administered with 1A8 or Clodrosome as compared to control depletions on the day of infection (Fig. 3a). We concluded that Clodrosome depleted primarily monocytic cells (macrophages and/or MO-MDSCs) and 1A8 depleted Ly6G^high^ iNOS^+^ PMN-MDSCs. The effect of depletion (a single versus multiple injections) on tumor growth in different groups was shown in Fig. 3b. We observed an initial tumor growth until day 7 in all of the treated groups although for the following next 3 days the growth patterns showed a variation with respect to different depletion regiments. While the tumors grew with a 1.6-fold increase in size in both of ly6G^+^ depletion groups (single or multiple), depletion of F4/80^+^ cells caused tumor regression. Although it was not significant, continuous depletion of ly6G^+^ cells further enhanced the growth until day 17. After day 17, an overall tumor regression was observed in all of the groups. These results suggested a role for iNOS^+^ cells as being part of an important effector mechanism during oncolysis, especially between days 7 and 10 p.i. In the next set of experiments, we used a selective iNOS inhibitor, L-NIL, to further confirm the involvement of PMN-MDSCs in tumor regression. Blocking of iNOS by its specific inhibitor showed an identical tumor growth induction trend between day 7 and 10 post infection as seen in the depletion experiments (Fig. 3c). Having established that neutrophilic iNOS^+^-MDSCs are participating in tumor regression, we next sought to confirm virus infection in the tumor without sacrificing the animals. Both LIVP 1.1.1 and its red-colored derivative GLV-2b372 were both similarly effective and tumors grew at an identical rate in HCT-116 and other tested models (data not shown). Fluorescence images obtained on day 3, 7 and 14 post-infection established a different kinetics of virus infection between the depletion groups (Fig. 3d). Clodrosome depletion of F4/80^+^, but not 1A8 depletion of iNOS^+^ cells, resulted in a massive infection of the tumor as early as 3 days p.i. as detected by the fluorescence light intensity of VACV-infected tumors. This rapid and extensive infection by VACV resulted in a better tumor regression in F4/80^+^ depleted-group compared to the iNOS^+^ depleted-group (Fig. 3b). The summary graph in Fig. 3e shows the relative fluorescence light intensity normalized to tumor volume. The depletion of F4/80^+^ cells every 3 days caused the most significant VACV replication and subsequent tumor regression starting from day 10. The data demonstrate that the cells responsible for restricting the tumor infection efficiency are monocytic rather than neutrophilic in origin. Overall, these results suggest that different myeloid cell subsets play different roles in the induction of antiviral immunity and antitumor immunity, and that these roles are not mutually exclusive.

### iNOS^+^ MDSCs have a cytotoxic effect on tumor cells due to NO production

**Fig. 4:**
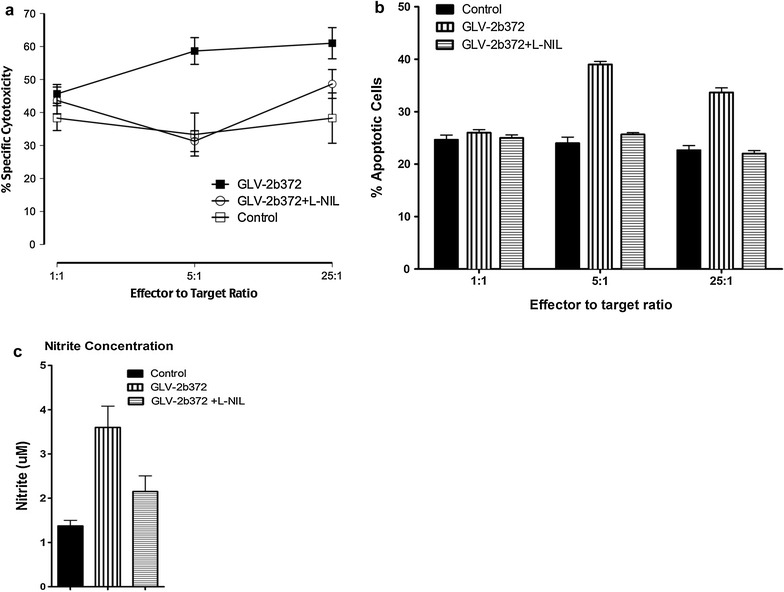
Functional change in iNOS+ MDSCs. **a** Total iNOS^+^ cells were harvested from the spleen of mice treated by VACV (10 d.p.i) (*filled square*) or untreated mice (*open square*). Isolated cells were mixed at the indicated ratios with HCT-116 target cells in a transwell system for 12 h. In addition, where indicated, 0.5 mM L-NIL was added to inhibit NO production. Cytotoxicity was measured by Alamar blue CTL assay and tumor killing was shown as % lysis. Experiments were performed in triplicate, n = 2. *Error bars* represent mean and SD. **b** In a separate experiment, HCT-116 cells isolated from the lower transwell chamber were analyzed by flow cytometry for apoptosis at the end of 12 h of culture period. The data is presented as % Annexin V^+^ cells. This is representative data out of two distinct experiments with similar results. **c** NO level was determined by nitrite measurement in the media. The culture supernatant obtained at 12 h of incubation period was used for the determination of nitrite level

Although the difference between iNOS^+^ PMN-MDSCs depleted groups and undepleted groups or L-NIL treated group and untreated groups were not statistically different, we observed a trend that suggested a potential therapeutic benefit by MDSCs’ high iNOS activity on tumor regression. To verify whether PMN-MDSCs cells were converted to tumor-killer cells in VACV treated mice, these cells were tested for in vitro cytotoxicity on tumor cells and NO production. Ly6G^+^ cells were purified from the spleen of tumor bearing mice on day 10 p.i. using Miltenyi anti-Ly-6G MicroBead kit and were co-cultured with HCT-116 cells in transwell system at various E:T ratios. The iNOS^+^ cell-mediated killing of target cells was significantly increased in the GLV-2b372-treated mice, but not in the control mice (Fig. 4a), in a dose-dependent manner. Although 1:1 E:T ratio did not affect the cytotoxicity, there was 1.76-fold increase in the cytotoxic activity of iNOS^+^-MDSC at a 5:1 E:T ratio. Cytotoxicity was due to NO secretion into culture media since addition of L-NIL reversed the killing of HCT-116 as it was detected by Alamar Blue CTL assay. To confirm that NO has an apoptotic effect on tumor cells, Annexin V staining was performed on HCT-116 cells isolated from the lower Transwell chamber and analyzed by flow cytometry. As it is shown in Fig. 4b, tumor cells co-incubated with iNOS^+^ MDSCs isolated from the spleen of GLV-2b372-treated mice had a higher apoptosis rate (39 ± 2.24%) as compared to the tumor cells co-cultured with iNOS^+^-MDSCs isolated from control mice (27 ± 1.69%). The cytotoxic effect of iNOS expression was also overlapped with significantly increased nitrite concentrations in the cultures, indicative of high NO production as assessed in the supernatant (Fig. 4c).

## Discussion

Recent advances in oncolytic immunotherapy suggest a significant role for this promising approach in the fast-expanding immuno-oncology field. It is acknowledged that host immunity contributes significantly to the oncolytic virus mediated antitumor response [39]. The innate immune system is primarily responsible for the initial containment of a virus, the viral infection, replication, spread and also the promotion of the initiation of an adaptive immune response against the viral pathogen. Accumulating data indicate that antiviral immunity adversely affects the efficacy of oncolysis by prematurely eliminating oncolytic viruses [40]. Paradoxically, the same antiviral response may have antitumor activities. Hence, during the infection, one of the mechanisms, iNOS-derived NO production by mature macrophages, is being activated to support the innate host defense against viruses and any other pathogenic microorganisms. We initially hypothesized that NO could be one of the factors that limits active spreading of the virus in the cancerous tissue. In contrast to our initial hypothesis, we observed that PMN-MDSCs were the main producer of NO through iNOS; NO provided a beneficial antitumor effect. We found that the recruitment of iNOS^+^-MDSCs at the VACV-infected tumor sites was significantly accelerated starting from 7 dpi. Despite the spike in their number and higher iNOS expression, virus titer was not reduced. The depletion of iNOS^+^-MDSCs as well as the administration of a selective iNOS inhibitor both induced tumor growth, implicating a role in tumor cell killing mechanism complementary to lysis by VACV. Finally, we showed that expression of iNOS was associated with a significant increase in NO production and tumor cell killing as demonstrated by in ex vivo experiments. This cell killing was transient and iNOS over expression returned to prior-infection levels between days 14–21. Whether this temporary effect can be further manipulated as an effective mechanism to control the tumor growth is currently unknown, but would be the subject of further studies.

GLV-1h68 is a recombinant isolate from the VACV LIVP strain, as described previously [41]. LIVP 1.1.1 is a less attenuated clonal isolate of the LIVP strain and has been recently tested as a more potent alternative to GLV-1h68 for clinical trials [26]. We previously analyzed the effect of a single administration of GLV-1h68, currently in human clinical Phase I/II trials as GL-ONC1, on the growth of HCT-116 colorectal cancer xenografts in nude mice [22]. This study suggested an early role of myeloid cells in triggering antitumor immune activity, although the mechanisms remained to be characterized.

In the tumor, NO was shown to be both friend and foe [5, 14]. It is believed that by producing variable levels of NO, iNOS can orchestrate various functions in different microenvironments [15]. Although observations about NO production as a result of oncolytic viruses cancer therapy are occasionally reported, the effect of the VACV infection on iNOS production by immature myeloid cells and the role of NO with regard to tumor regression and virus survival in vivo have not been studied for this sole purpose before. Therefore, in this study, we focused mainly on the action mechanism of iNOS^+^-MDSC as a response to VACV therapy and investigated VACV induced-NO production and its effect on virus replication efficiency and tumor regression.

Antiviral effects of NO, produced from phagocytic cells such as neutrophils and macrophages are well known for some viruses, typically DNA viruses [24]. However, NO production does not necessarily correlate with viral clearance in the infected tumor, as our data suggested. NO-induced oxidative injury may be attributable to pathogenesis of infection with certain viruses that are resistant to the direct antiviral actions of NO, as observed with vaccinia virus.

The main hallmark of MDSCs is their tumor promoting capacity. Various approaches have been tested to eliminate their presence, block their accumulation, minimize their immunosuppressive function and induce their differentiation into mature phenotype [42]. The accumulation of granulocytic MDSCs following oncolytic viral therapy in mice has been observed in many different tumor models [18, 32–34]. Furthermore, many viruses are not oncolytic but are still capable of creating an inflammatory environment and promoting the induction and accumulation of iNOS^+^ cells [43, 44]. We believe that there are no discrepancies between activated PMN-MDSC recruited in mice bearing xenograft tumors versus syngeneic tumors. A basic understanding of the role of these cells in response to VACV therapy in mice establishes a framework for future studies in clinical trials. It is highly probable that inflammatory mediators generated during infection contribute to the recruitment of MDSCs. In previous studies other researchers and we have done profiling on mouse immune-related genes and showed a drastic change in the transcriptional increase in the pro-inflammatory genes. Among them, MCP-1 (CCL-2), IL-1b, IL-6, TNF-α were especially shown to be involved in the recruitment of immature cells into tumor microenvironment [18, 45, 46].

Two recent observations about the function of MDSCs in virus-treated tumors highlight distinct and important aspects of these suppressors. In one of these studies, it has been shown that NK cell response to VACV infection is negatively regulated by granulocytic MDSCs [20]. In a more recent study, Eisenstein et al. exploring MDSCs as a vehicle to transport vesicular stomatitis virus (VSV), reported the differentiation of MDSCs towards M1-like phenotype following the encounter of MDSC with virus in tissue culture plate [17]. Our extensive in vivo analysis not only parallel their findings, but also demonstrate that in situ conversion of tumor-promoting MDSCs into tumor-killing cells by oncolytic VACV is an important effector mechanism which directly contributes to the therapeutic effect of VACV. A combination of all of these results is particularly important because it indicates the multiple immune modulatory effects of oncolytic vaccinia virotherapy through MDSCs. It is likely that iNOS^+^-MDSC can inhibit an antiviral immune response by blocking NK cells and suppressing CD8 T-cell activity during oncolysis. On the other hand, we cannot rule out the possibility that the accumulation of iNOS^+^-MDSC could also potentially hamper the subsequent development of an antitumor T-cell response. We and other researchers have shown that NO production was responsible for the suppression of T-cells [30–47]. However, our data indicated that the iNOS-upregulation is short-lived and possibly subsided by the time CTLs start infiltrating the tumors. Although we hypothesize that timing of CTL infiltration and iNOS-upregulation do not overlap, further studies in syngeneic models are needed to delineate the exact kinetic.

The cells responsible for preventing the effective infection of the tumor following r.o. injection, appeared to be monocytic rather than neutrophilic cells. Different subsets of myeloid cells (monocytic and neutrophilic) have been reported during VACV infection [48]. In one of these studies, Ly6C^+^ phagocytic cells prevented spreading of virus and their depletion by Clodrosome enhanced the replication at the site of infection. Interestingly, the lesion size was increased in the tissue of mice depleted of ly6G^+^ subsets. The response observed in our depletion and infection studies combined, did not differ from this finding. Clodronate liposome depletion was used as an alternative to antibody depletion targeting specifically the Ly6G^+^ component. However, Clodrosome did not deplete iNOS^+^ cells to the same extent as it is with the ly6G^+^ antibody but instead depleted exclusively F4/80+ monocytic cells. This caused a rapid and increased replication of VACV as it has been shown before [49].

The heterogeneous composition of MDSCs under different conditions such as inflammatory/neoplastic in addition to the limited surface markers affected by different level of immaturity make comparison of different subsets with normal counterpart unlikely [50]. Therefore, we do not know whether the PMN-MDSC within the tumors are actually neutrophils or the immature precursors of granulocytic MDSCs which include neutrophils at earlier stage of maturation and/or N1 versus N2 polarized tumor-associated neutrophils (TAN) phenotypes. It is acknowledged that similar tumor cell cytotoxicity was observed with tumor-activated neutrophils during tumor progression. Interestingly, neutrophils can also acquire immunosuppressive activity if activated [51]. Although they were initially seen as cells with a sole function to eliminate invading microorganisms, growing evidence point out them as potent regulatory cells expressing numerous effector molecules. Taken together, there seems to be a significant functional overlap between PMN-MDSC and neutrophils in the cancer microenvironment and elevated iNOS activity is instrumental in both immunosuppression and tumoricidal activity, making these two populations not only phenotypically but also functionally equivalent.

## Conclusions

Clearly, a better understanding of the mechanisms of tumor regression by oncolytic viruses is needed to allow the induction of effective antitumor immunity resulting in a better therapeutic outcome. While implicated in few studies, a detailed understanding of how iNOS^+^ MDSCs modulate the antitumor immune response during an oncolytic virus treatment remains under-explored. To the best of our knowledge, this is the first report that shows in vivo synergistic tumor cell killing by VACV and MDSCs. If this ability of MDSCs to switch from tumor-promoting to tumor-killing could be effectively utilized, it may significantly enhance the therapeutic potential of oncolytic virus therapy.

## Abbreviations

VACV: vaccinia virus
MSDC: myeloid-derived suppressor cell
iNOS: inducible nitric oxide synthase
NO: nitric oxide
LPS: lipopolysaccharides
PMN: polymorphonuclear
MO: monocytic
Dpi: days post-infection
MFI: mean fluorescence intensity
L-NIL: N6-(1-iminoethyl)-l-lysine dihydrochloride
